# Particulate Matter Exposure During Oocyte Maturation: Cell Cycle Arrest, ROS Generation, and Early Apoptosis in Mice

**DOI:** 10.3389/fcell.2020.602097

**Published:** 2020-11-26

**Authors:** Yu-Jin Jo, Seung-Bin Yoon, Byoung-Jin Park, Sang Il Lee, Ki Jin Kim, Se-Yong Kim, Minseong Kim, Jun-Ki Lee, Sang-Yong Lee, Dong-Ho Lee, Taeho Kwon, Yeonghoon Son, Ja-Rang Lee, Jeongwoo Kwon, Ji-Su Kim

**Affiliations:** Primate Resources Center (PRC), Korea Research Institute of Bioscience and Biotechnology (KRIBB), Jeongeup, South Korea

**Keywords:** particulate matter, oocyte maturation, cell cycle arrest, polar body extrusion, time-lapse microscopy

## Abstract

Particulate matter (PM) is a general atmospheric pollutant released into the air by an anthropogenic and naturally derived mixture of substances. Current studies indicate that fine dust can result in different health defects, including endothelial dysfunction, asthma, lung cancer, cardiovascular diseases, uterine leiomyoma, deterioration in sperm quality, and overall birth impairment. However, the most prominent effects of PM_10_ (diameter < 10 μM) exposure on the female reproductive system, especially with respect to oocyte maturation, remain unclear. In the present study, maturing mouse oocytes were treated with PM_10_ and the phenotypes of the resulting toxic effects were investigated. Exposure to PM_10_ led to impairment of maturation capacity by inducing cell cycle arrest and blocking normal polar body extrusion during *in vitro* maturation and activation of fertilization of mouse oocytes. Additionally, defects in tubulin formation and DNA alignment were observed in PM_10_-treated oocytes during metaphase I to anaphase/telophase I transition. Moreover, PM_10_ induced reactive oxygen species generation, mitochondrial dysfunction, DNA damage, and early apoptosis. Taken together, these results indicate that PM_10_ exposure leads to a decline in oocyte quality and affects the subsequent embryonic development potential of mammalian oocytes.

## Introduction

Particulate matter (PM) is a general atmospheric pollutant introduced into the air by an anthropogenic and naturally derived mixture of substances. These substances exist in various shapes and sizes and consist of a wide range of chemicals such as Cd, Ni, and Pb, which may vary across regions. Prior evidence indicates that PM of a minute size (diameter < 2.5 μM) can penetrate deep into the alveoli of lungs and can potentially enter the blood circulation, crossing the air–blood barrier; it is even capable of reaching other organs (Duan et al., 2017). High concentrations of NO_2_, SO_2_, and O_3_ in PM_10_ have been shown to increase infertility rates and incidence of miscarriage (Checa Vizcaino et al., 2016). The various intracellular toxic effects generated by PM_10_ are primarily due to an elevation in reactive oxygen species (ROS) levels in addition to the apoptosis resulting from DNA damage and hydroxyl radicals induced by the high iron content in PM_10_ (Alfaro-Moreno et al., 2002).

In human studies, long-term exposure to PM was found to result in the exacerbation of various diseases, including asthma (Evans et al., 2014), chronic obstructive pulmonary disease, lung cancer (Hamra et al., 2014), and cardiovascular diseases (Huttunen et al., 2012). Furthermore, PM is associated with an increased incidence of postnatal diseases, such as early age leukemia (Andrade et al., 2014), bronchiolitis in infants (Lanari et al., 2015), and childhood asthma (Perera et al., 2009). Other studies have also reported the effects induced by PM, including embryonic stage cellular toxicity and teratogenicity, in various mammalian species; moreover, its impact on human fetal development has also been documented (Manzo et al., 2010).

PM_10_ and PM_2_._5_ have been shown to accelerate infertility by impacting germ cell development. When present as ambient air pollutants, PM_10_ and PM_2_._5_ cause a decline in sperm quality, particularly in regard to concentration, motility, and abnormal morphology following a decrease in testosterone levels (Hammoud et al., 2010; Radwan et al., 2016). In females, cumulative average PM_2_._5_ levels heighten the risk of uterine leiomyoma (Mahalingaiah et al., 2014). In addition, exposure to high concentrations of atmospheric particulate matter affects the overall pregnancy and birthing process, with adverse effects such as reduced birth weight (Kumar, 2016; Wylie et al., 2017) or delayed growth (Liu et al., 2007), abortion (Enkhmaa et al., 2014), and stillbirth (Siddika et al., 2016). Exposure to PM_10_, NO_2_, and CO leads to a consequent low pregnancy rate in the IVF cycle following a decreased probability of intrauterine implantation (Choe et al., 2018). In zebrafish embryos, PM exposure triggers an increase in mortality rate and malformations, with a concomitant reduction in hatching rate and body length caused by an induction of ROS generation and autophagy (Zhang et al., 2018). As prior research demonstrates, PM exposure may disrupt the overall duration of the developmental stage, including the meiotic process in the ovary as well as early embryogenesis and fetal growth during pregnancy. However, the effects of PM and the underlying mechanism of cellular toxicity during female oocyte maturation remain unclear.

In the present study, the toxic effect of PM_10_ during mouse oocyte maturation was evaluated. The rates of oocyte maturation, cell cycle arrest, and maturation-promoting factor (MPF) degradation were assessed at different concentrations of PM_10_. Spindle formation and DNA alignment at the metaphase I (MI) stage oocyte were examined. Furthermore, DNA damage, ROS levels, and mitochondrial function were analyzed following PM_10_ exposure to maturing oocytes. Based on our results, the role of PM as a risk factor in air pollution-impacted environments was hypothesized; we also proposed that female infertility by impacting the subsequent embryonic development potential of mammalian oocytes.

## Materials and Methods

### Oocyte Collection, *in vitro* Maturation, and PM Treatment

All animal studies were approved and conducted according to the guidelines of the Animal Research Committee of Korea Research Institute of Bioscience and Biotechnology (KRIBB-AEC-19126). Germinal vesicle (GV) intact oocytes were collected from the ovaries of 6–8-week-old CD-1 mice and cultured in M16 medium (Sigma-Aldrich, St. Louis, MO) with an overlaying mineral oil layer at 37°C under 5% CO_2_.

For PM_10_ (#ERM-CZ120; Sigma Aldrich) treatment, oocytes were cultured in media supplemented with concentrated PM_10_. Before the experiment, PM_10_ was dissolved in M16 medium according to the experimental concentration (1, 5, and 10 mg/mL), and pre-warmed at 37°C at least overnight. Thereafter, only completely dissolved PM_10_ was obtained by filtration through a 0.22-μm filter. Oocytes were then cultured in PM, which was 100% soluble.

### Parthenogenetic Activation

As described previously (Jo et al., 2019), mature oocytes that underwent cytokinesis and first polar body (PB) extrusion were activated in Ca^2+^-free Chatot–Ziomek–Bavister medium (Chatot et al., 1989) supplemented with 10 mM SrCl_2_ and overlaid with paraffin oil at 37°C under 5% CO_2_ conditions for 4 h. Oocytes were washed in potassium simplex optimization medium (KSOM; MilliporeSigma, Burlington, MA), and incubation continued at 37°C. To check the activation rate, oocytes that extruded the second PB were counted.

### Microinjection and Time-Lapse Microscopy

Time-lapse imaging was performed for control and PM_10_-treated oocytes as described previously (Jo et al., 2016). mRNA was microinjected into the cytoplasm of a GV-stage oocyte using a microinjector (Eppendorf, Hamburg, Germany) for labeling cyclin B, tubulin, or DNA. For visualizing the MPF degradation pattern, cyclin B-GFP mRNA (300 ng/μL) was injected; for visualizing the chromosomes and spindle, H2B-mCherry mRNA (200 ng/μL) and tubulin-GFP mRNA (200 ng/μL) were injected, respectively. mRNA-injected oocytes were arrested for 3 h at the GV stage in M16 medium supplemented with 4 μM milrinone, with an overlaying mineral oil layer, at 37°C under 5% CO_2_ for subsequent mRNA expression analysis. Prior to live-cell imaging, oocytes were washed in M16 without milrinone five times and transferred into fresh M16 medium. Images were acquired at intervals of 5 min for 12 h using the EVOS M7000 Imaging System (Thermo Fisher Scientific, Waltham, MA), which was maintained at 37°C under 5% CO_2_ conditions.

### Western Blotting

Fifty oocytes in each group were washed twice with PBS, lysed in 20 μL lysis buffer (20 mM HEPES, 150 mM NaCl, 2 mM EGTA, 1 mM EDTA, 20 mM glycerol phosphate, 1% Triton X-100, 4% SDS, 14.4 mM 2-mercaptoethanol, and 0.04% bromophenol blue), and then boiled at 100°C for 5 min. Protein samples were separated on a 10% SDS-polyacrylamide gel and transferred to a nitrocellulose membrane (Millipore, MA, United States). The membrane was blocked in TBS containing 0.25% Tween 20 (TBST) and 5% BSA for 1 h, rinsed in TBST, and probed with an anti-cyclin B1 (1:1,000; #ab72; Abcam, Cambridge, United Kingdom), anti-CDK1 (1:500; #ab18; Abcam), and anti-α-tubulin (1:2000; #T5168; Sigma-Aldrich) antibodies at 4°C overnight. The blot was washed with TBST and subsequently incubated with horseradish peroxidase-conjugated secondary antibody. The membranes were visualized using an enhanced chemiluminescence detection reagent (SuperSignal West Pico Plus; Thermo Fisher Scientific) according to manufacturer’s instructions. The protein level were semi-quantified via densitometry using ImageJ software (version 1.47; NIH, Bethesda, MD; http://imagej.nih.gov/ij).

### Real-Time RT-PCR

Poly(A) mRNAs were extracted from 50 oocytes using the Dynabeads mRNA Direct Micro Purification Kit (#61021, Thermo Fisher Scientific) according to the manufacturer’s protocol. Briefly, samples were lysed in 100 μL of lysis/binding buffer at room temperature for 10 min, after which 20 μL of Dynabeads Oligo (dT)25 was added to each sample. The beads were hybridized for 5 min and then separated from the binding buffer using a DynaMag-2 Magnet (Thermo Fisher Scientific). Bound poly(A) mRNAs and beads were washed with buffers A and B, and then separated by the addition of 10 μL of 10 mM Tris-HCl buffer. Poly(A) mRNAs were reverse-transcribed in a 20-μL reaction volume containing oligo(dT)20 primer, 5X RT buffer (containing 25 mM Mg^2+^), 10 U RNase inhibitor ReverTra Ace (Toyobo, Osaka, Japan), and a 10 mM mixture of dNTPs. Secondary RNA structure was denatured by incubating at 42°C for 60 min to facilitate cDNA generation. The reaction was terminated by incubation at 99°C for 5 min. The resulting cDNA was used as a template for PCR amplification with PowerUp SYBR Green Master Mix (Thermo Fisher Scientific) on a StepOnePlus Real-Time PCR System (Thermo Fisher Scientific). The PCR cycling conditions were as follows: 95°C for 30 s, 60°C for 30 s, and 72°C for 30 s, (40 cycles)followed by extension at 72°C for 5 min. A housekeeping gene (18S RNA) was used as an internal standard for each group. Mouse primers were designed using Primer3 (http://bioinfo.ebc.ee/mprimer3). The primer sequences are listed in Supplementary Table S1.

### Immunostaining

Oocytes were fixed in 3.7% paraformaldehyde dissolved in polyvinyl alcohol in PBS (PVA-PBS) for 30 min at room temperature or overnight at 4°C. For permeabilization, fixed oocytes were transferred into 0.5% Triton X-100 for 1 h at room temperature. After blocking in 1% BSA in PBS for 1 h, oocytes were stained with the primary antibodies rabbit anti-phospho-Histone H2A.X (1:200; #9718T; Cell Signaling Technology, Danvers, MA) and mouse anti-pericentrin (1:200; #611814; BD Biosciences, Franklin Lakes, NJ) at 4°C overnight. After washing the oocytes three times with PVA-PBS, they were labeled with secondary antibodies Alexa Fluor 594 goat anti-rabbit and Alexa Fluor 488 goat anti-mouse (1:200; #A11012; #A11001, respectively; Thermo Fisher Scientific) at room temperature for 2 h. For spindle staining, the oocytes were incubated with mouse monoclonal FITC-conjugated anti-α-tubulin antibody (1:200; Sigma-Aldrich) for 2 h at room temperature. Stained oocytes were washed extensively with PVA-PBS and incubated with Hoechst 33342 (10 μg/mL; #B2261; Sigma-Aldrich) for 20 min. After washing, the samples were mounted on glass slides in Vectashield containing DAPI (Vector Laboratories, Burlingame, CA) and observed under a fluorescence microscope (Zeiss, Oberkochen, Germany).

### Comet Assay and Annexin V Staining

Comet assays were conducted using the OxiSelect Comet Assay Kit (#STA-350; Cell Biolabs, San Diego, CA) according to manufacturer’s instructions. The comet assay tail length was calculated using ImageJ. To investigate the rate of early apoptotic oocytes, annexin V staining was conducted for 20 min in the dark after washing the oocytes in PVA-PBS. According to the protocol, oocytes were incubated in 90 μL binding buffer containing 10 μL Annexin V-FITC (Vazyme Biotech Co., Ltd., Nanjing, China). After washing three times with PVA-PBS, oocytes were mounted on a slide glass and the fluorescence signal was immediately examined using a fluorescence microscope (DMi8; Leica Microsystems, Wetzlar, Germany).

### ROS, Glutathione (GSH), and Cathepsin B Staining

To determine the levels of intracellular ROS and GSH in living oocytes, oocytes were stained for 30 min in PVA-PBS containing 10 μM 2´,7´-dichlorodihydrofluorescein diacetate (H_2_DCFDA; Invitrogen, Carlsbad, CA) or 4-chloromethyl-6,8-difluoro-7-hydroxycoumarin (CMF_2_HC; Invitrogen), respectively, at 37°C. After incubation, oocytes were washed three times and then transferred into PVA-PBS droplets and covered with mineral oil. Fluorescence signals were observed using a fluorescence microscope (DMi8). ROS and GSH levels were quantified by determining the fluorescence intensity in the cytoplasm of the oocytes using ImageJ. For measuring cathepsin B activity, oocytes were stained according to the Magic Red Cathepsin B Assay Kit (Immunochemistry Technologies LLC, Bloomington, MN) protocol. The same procedure was used for all replicated groups. The obtained intensity data were normalized by subtracting the background intensity from each oocyte size and dividing by the mean value of the control groups. All experiments were repeated at least three times and the number of analyzed oocytes are indicated in the figures.

### JC-1 and MitoSOX Staining

For JC-1 or MitoSOX staining, Metaphase II (MII)-stage oocytes in the control and PM-treated groups were incubated in PVA-PBS medium containing 2.5 #x00B0;M 5,5´,6,6´-tetrachloro-1,1´,3,3´-tetraethyl-imidacarbocyanine iodide (JC-1; cat #M34152; Invitrogen) or 5 mM MitoSOX red (Life Technologies, Carlsbad, CA), respectively, at 37°C in 5% CO_2_ for 30 min. After the washing step, oocytes were transferred into a PVA-PBS droplet for imaging using a fluorescence microscope (DMi8). The mitochondrial membrane potential (ΔΨm) was determined by calculating the ratio of the fluorescence intensity of the activated mitochondria (J-aggregates; red)] to that of the J-monomer (green). The relative intensity of MitoSOX staining was quantified by measuring the mean value in ImageJ at the same time as exposure to the fluorescence laser.

### Statistical Analysis

All experiments in the present study were replicated at least three times for each treatment or imaging study. Statistical analyses were performed using Welch’s *t*-test, Pearson’s chi-square test, Fisher’s exact test, or analysis of variance, followed by Tukey’s multiple comparisons of means with R software (R Development Core Team, Vienna, Austria). Data are expressed as the mean ± SEM, and differences were considered statistically significant at *P* < 0.05.

## Results

### PM_10_ Induces Defects in Oocyte Maturation and Activation of Mature Oocytes

**FIGURE 1:**
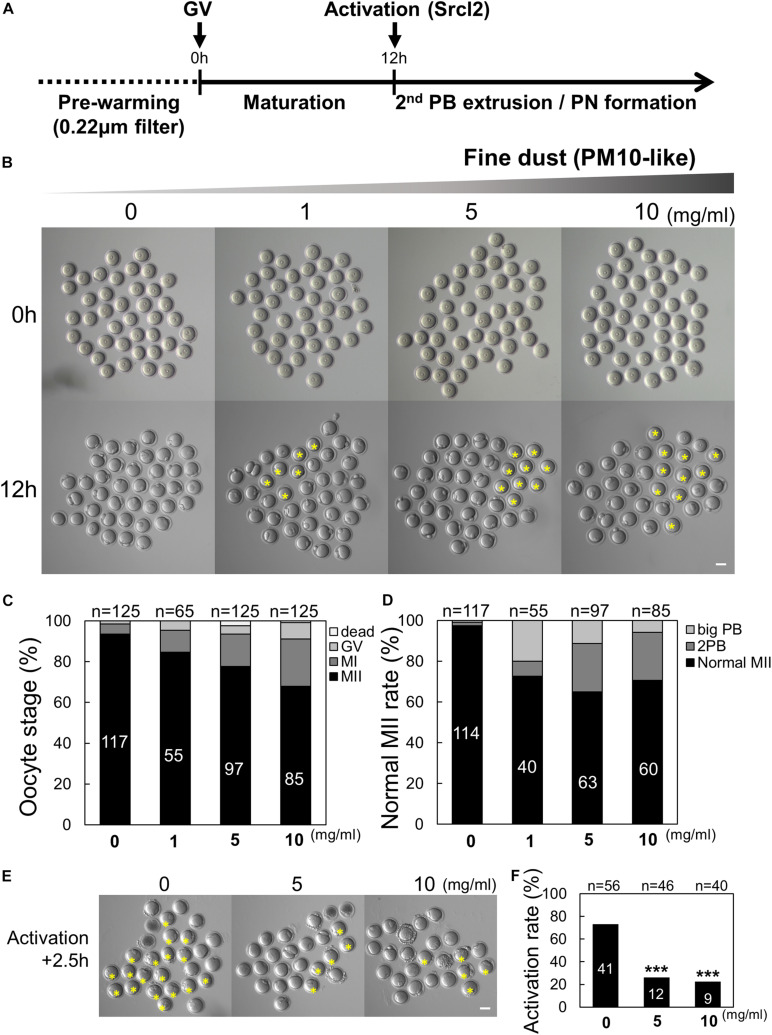
Effect of particulate matter (PM_10_) exposure on mouse oocyte maturation and activation rate. **(A)** Timeline for the experimental schedule of PM_10_ treatment. **(B)** Germinal vesicle (GV) oocytes were cultured at the indicated PM_10_ concentrations for 12 h. PB (polar body) extrusion failed after PM_10_ exposure. The yellow asterisks indicate oocytes arrested at MI. **(C)** Percentage of oocytes in each stage after maturation (12 h) of control (0 mg/mL) and PM_10_-treated oocytes. **(D)** MII rate was calculated as the formation of one normal PB in the oocyte. **(E)** Impairment of activation after PM_10_ exposure during *in vitro* maturation. Yellow asterisks indicate failed second PB extrusion in the oocyte at 2.5 h after Srcl2 treatment. **(F)** Activation rate of control and PM_10_-treated MII oocytes. ***P < 0.001. *n* indicates the total number of cultured oocytes. Scale bars = 50 μm.

To investigate the effect of PM_10_ on asymmetric cell division and first PB extrusion, we treated maturing oocytes with PM_10_. To our knowledge, there are no prior studies on PM_10_-induced defects in mammalian oocytes; thus, we evaluated the effect of PM_10_ exposure at different concentrations as shown in Figure 1A. The PB extrusion rate of oocytes decreased and the number of metaphase I-arrested oocytes increased in a PM_10_ dose-dependent manner (Figures 1B,C). In addition to failed meiotic cell division, PM_10_-treated mature oocytes extruded large PBs, or there was a segregation into two PBs, and showed a decreased MII formation rate comparison with that of control oocytes (Figure 1D).

The normal second PB extrusion of mature oocytes is an important indicator of whether oocytes are capable of fertilization. Therefore, the activation rate was investigated by counting the second PB extruded by oocytes in control and PM_10_-treated oocytes. After 2.5 h, the percentage of activated oocytes significantly decreased in the PM_10_ treatment groups (5 and 10 mg/mL) compared with the control (Figures 1E,F). Taken together, these results indicate that exposing oocytes to PM_10_ adversely affects cytokinesis as well as normal PB formation during maturation and activation, even during the first meiotic division.

### PM_10_ Exposure Induces Cell Cycle Arrest During MI–ATI Transition by Delaying Cyclin B Degradation

**FIGURE 2:**
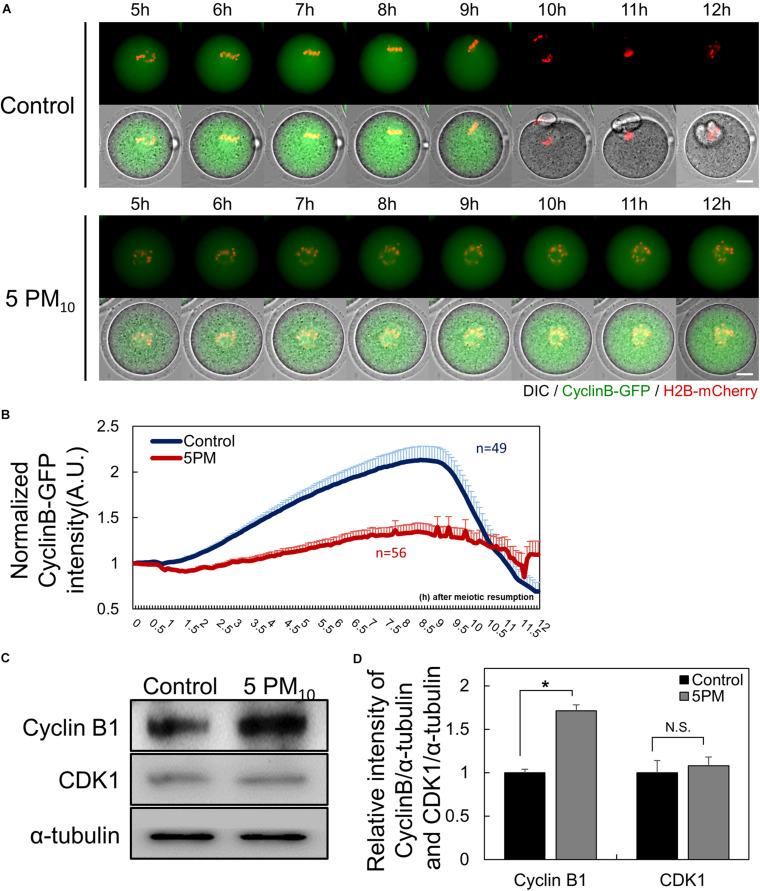
PM_10_ treatment delays anaphase transition timing during mouse oocyte maturation. **(A)** Time-lapse microscopy of maturing control (0 mg/mL) and PM_10_ (5 mg/mL)-treated oocytes. Cyclin B, green; DNA, red. The full time lapse recording can be seen in Supplementary Movie 1 and 2. Scale bar = 20 μm. **(B)** The fluorescence intensity of cyclin B-GFP normalized at maturation of oocytes undergoing meiotic division. Data are presented as the mean ± SEM. Experimental replicate three times. Sample sizes *(n)* are indicated. **(C)** Western blotting of cyclin B and Cdk1 (MPF subunits) in ATI stage oocytes. Oocytes were sampled at 9 h after maturation. **(D)** Relative protein expression levels of cyclin B and CDK1. Cyclin B protein was degraded at the ATI stage in control oocytes, but was maintained in the PM_10_ group. The experiment was performed three times. *P = 0.0184. N.S., not statistically significant (*P* > 0.05).

Given that cytokinesis failed in PM_10_-treated oocytes, we evaluated MPF levels to determine the effect of PM_10_ exposure on MI–ATI transition during oocyte maturation. Time-lapse microscopy was performed to monitor the MPF degradation pattern after injecting cyclin B-GFP mRNA (Figure 2A). The intensity of cyclin B-GFP rapidly decreased in the control oocytes at 9.5 h during the anaphase–telophase transition (Figures 2A,B, and Supplementary Movie 1). The decrease in cyclin B-GFP intensity was delayed or not observed in the PM_10_ exposure group, although some oocytes with PB extrusion underwent cyclin B degradation (Figures 2A,B, and Supplementary Movie 2). Also, PM_10_ exposure oocytes failed accumulation of cyclin B during the pre-MI (Figure 2B).

Next, we measured the protein levels of cyclin B1 and CDK1, which are MPF subunits. Oocytes were sampled at 9 h after meiotic resumption to determine cyclin B1 protein levels. In PM_10_-treated oocytes, cyclin B1 was not degraded, whereas protein levels in the control oocytes were decreased, as evidenced by western blotting (Figure 2C). Moreover, the relative intensity of cyclin B1 protein levels normalized to α-tubulin was significantly increased in the PM_10_-treated group compared with the control (Figure 2D). The relative intensity of CDK1, the non-degradable subunit of MPF, did not significantly differ between the control and PM_10_-treated groups (Figure 2D). Thus, the results indicate that PM exposure disrupts the accumulation and/or degradation of cyclin B1 during pre-MI and MI–ATI transition.

### PM_10_ Exposure Results in Abnormal Spindle Formation and Impairs Chromosome Alignment

**FIGURE 3:**
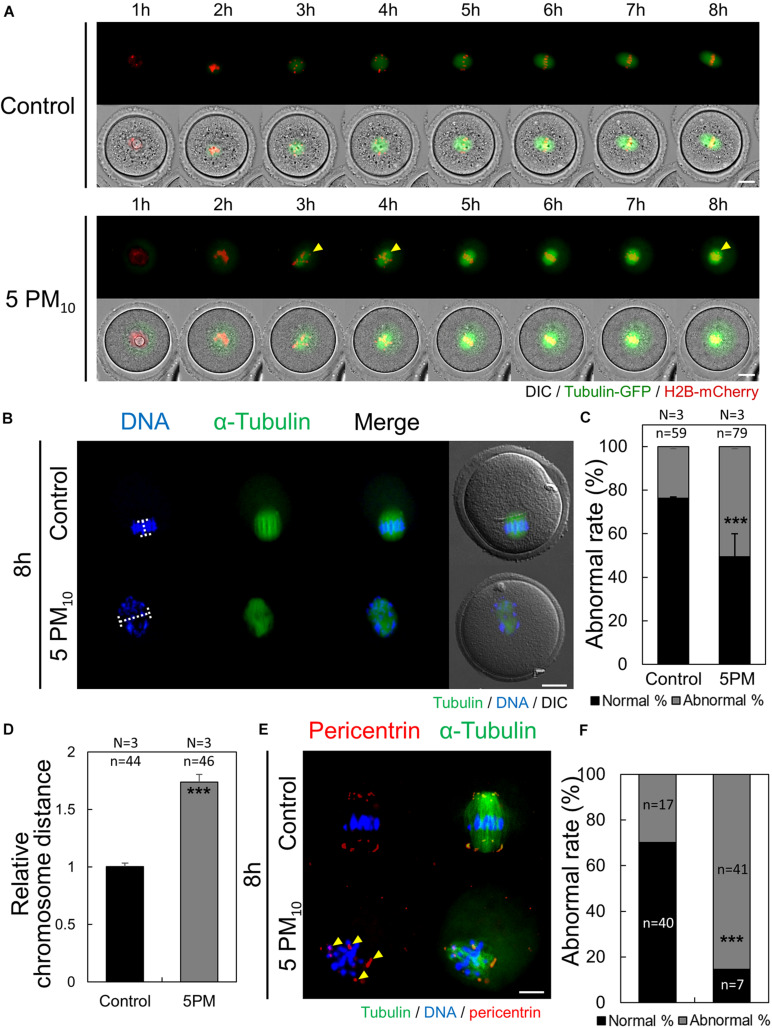
PM_10_ exposure induces defects in spindle assembly and chromosome alignment in mouse oocytes. **(A)** Time-lapse microscopy of maturing control and PM_10_-treated oocytes. The Spindle, green fluorescence. Yellow arrows indicate misaligned chromosome. **(B)** Defective spindle morphology and chromosome alignment are shown. Oocytes were fixed at 8 h after meiotic resumption. Green; spindle, and DNA was counterstained using Hoechst 33342 (blue). **(C)** The percentage of abnormal spindle formation. **(D)** Quantification of the distance between chromosomes. **(E)** Localization of pericentrin 8 h after meiotic resumption. Yellow arrows indicate scattered pericentrin. **(F)** Quantification of the abnormal rate of mislocalized pericentrin. Experimental replicate (N) and *n* sample size are indicated. ***P < 0.001. Scale bars = 20 μm.

We then investigated whether the toxic effects of PM impair meiotic spindle formation during the early stages of oocyte maturation. Oocytes were injected with tubulin-GFP and H2B-mcherry to label microtubules and chromatin, respectively, and time-lapse microscopy was performed from meiotic resumption to the MI stage (0–8 h). PM_10_ exposure disrupted barrel-shaped spindle formation and caused DNA misalignment (Figure 3A, yellow arrows; Supplementary Movies 3,4). However, after meiotic resumption in the control group, microtubules accumulated at 3–5 h, chromosomes aligned at the oocyte center at 6 h, and the normal meiotic spindle formed.

**FIGURE 4:**
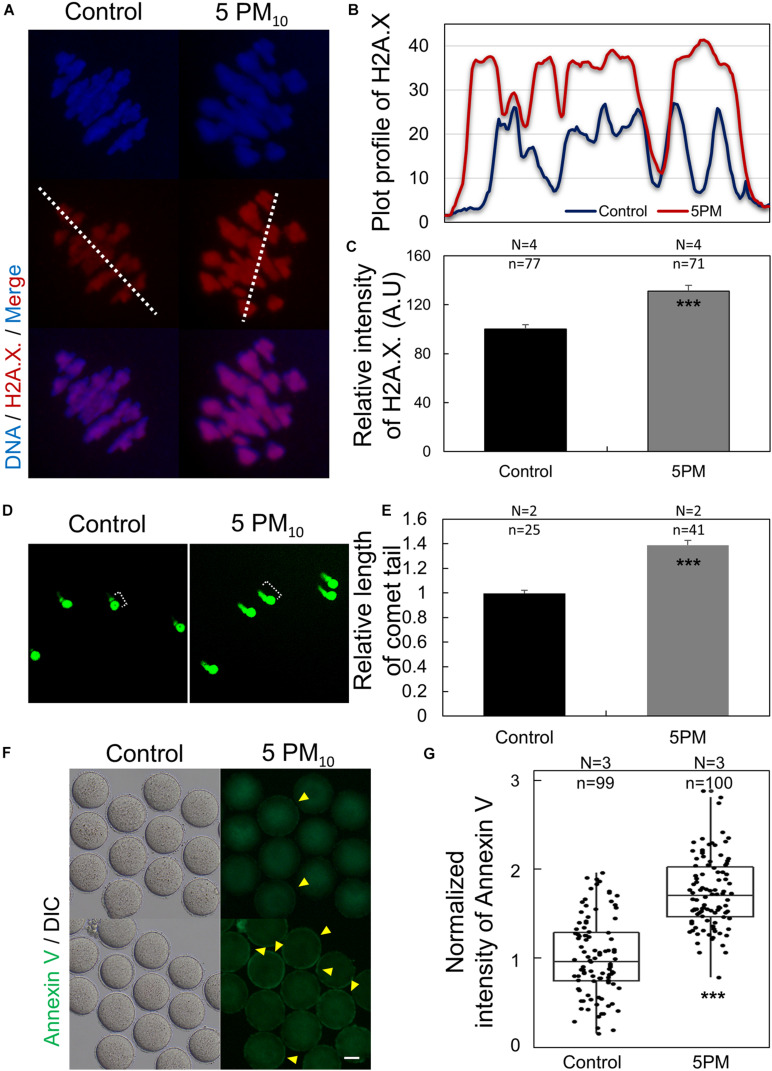
PM_10_ exposure induces DNA damage and early apoptosis in mouse oocytes. **(A)** DNA damage after PM_10_ treatment in chromosomes of an MI stage oocyte. **(B)** Plot profile of H2A.X. fluorescent signal as indicated by the white line in **(A)**. **(C)** Relative fluorescent intensity levels of H2A.X. **(D)** Comet assay for assessing DNA damage in MI stage oocytes. Control oocytes show slight DNA damage, whereas PM_10_-exposed oocytes show notable DNA damage. **(E)** Relative tail length of control and PM_10_-treated groups. **(F)** Early apoptosis evaluated by annexin V staining of zona-free MI stage oocytes. Yellow arrowheads indicate annexin V-positive oocytes. Scale bar = 40 μm. **(G)** Percentage of annexin V-positive control and PM_10_-treated oocytes. Experimental replicates (N) and *n* sample sizes are indicated in **(C)**, **(D)**, and **(F)**. ***P < 0.001.

To calculate the rate of abnormal spindle formation in the control and PM_10_-treated groups, α-tubulin immunostaining was performed at 8 h after meiotic resumption. Compared with the control oocytes that formed normal barrel-shaped bipolar spindles at the MI stage (Figure 3B), PM_10_-treated oocytes had significantly increased rates of abnormal meiotic spindle formation, showing scattered or monopolar morphology (Figures 3B,C). The ratio of misaligned chromosomes in MI-stage control and PM_10_-treated oocytes was measured by determining the aligned chromatin width (Figures 3B,D, 4A). PM_10_-exposed oocytes exhibited significantly increased inter-chromatin distances compared with that of control (Figure 3B, white line; Figure 3D). These results indicate that PM_10_ induces incomplete meiotic spindle formation and chromosome misalignment. During oocyte maturation, the meiotic spindle is generated from the acentriolar microtubule-organizing center (aMTOC) localized at the spindle pole (Schuh and Ellenberg, 2007). Thus, to elucidate the spindle defects and chromosome misalignment induced by PM exposure, we investigated pericentrin, a marker of aMTOC. Pericentrin was localized at both ends of the meiotic spindle in control oocytes, as confirmed previously (Clift and Schuh, 2013; Adhikari and Liu, 2014; Lee et al., 2018). However, in PM_10_-exposed oocytes, pericentrin was scattered within the cytoplasm (Figures 3E,F). Thus, PM exposure impairs aMTOC localization, resulting in abnormal spindle formation and chromosome misalignment.

### PM_10_ Treatment Triggers DNA Damage and Early Apoptosis During Mouse Oocyte Maturation

To examine whether PM_10_ induces DNA lesions during oocyte maturation, we determined phosphorylated H2A.X levels in the control and PM_10_-exposed oocytes via immunostaining. γH2A.X was abundant in the chromatin of PM_10_-treated oocytes (Figure 4A). Plot profile data revealed that the γH2A.X fluorescence signal was accumulated and amplified, and consequently stronger in the PM_10_-treated oocytes than in the control (Figure 4B). Moreover, the normalized intensity of γH2A.X was significantly increased in the PM_10_ group compared with controls (Figure 4C). DNA comet assay results also showed that DNA damage was markedly increased, with a significantly longer comet tail length in the PM_10_-exposed oocytes than in controls (Figures 4D,E). These results indicate that exposure to PM_10_ triggers DNA damage, which may result in cell apoptosis. To test this, we performed annexin V-FITC staining and found that PM_10_ exposure induced early apoptosis in oocytes (Figures 4F,G). To obtained clear signals, we removed the zona pellucida of the oocytes using Tyrode’s solution. Annexin V-positive oocytes, which displayed green fluorescence in the external cell membrane of oocyte regardless of zona pellucida presence, were markedly increased in the PM_10_-treated group compared with control (Figure 4G). These results indicate that PM_10_ exposure triggers chromosome and DNA damage, resulting in early apoptosis.

### PM_10_ Exposure Induces ROS Generation, Decreases GSH Levels, and Augments Cathepsin B Activity

**FIGURE 5:**
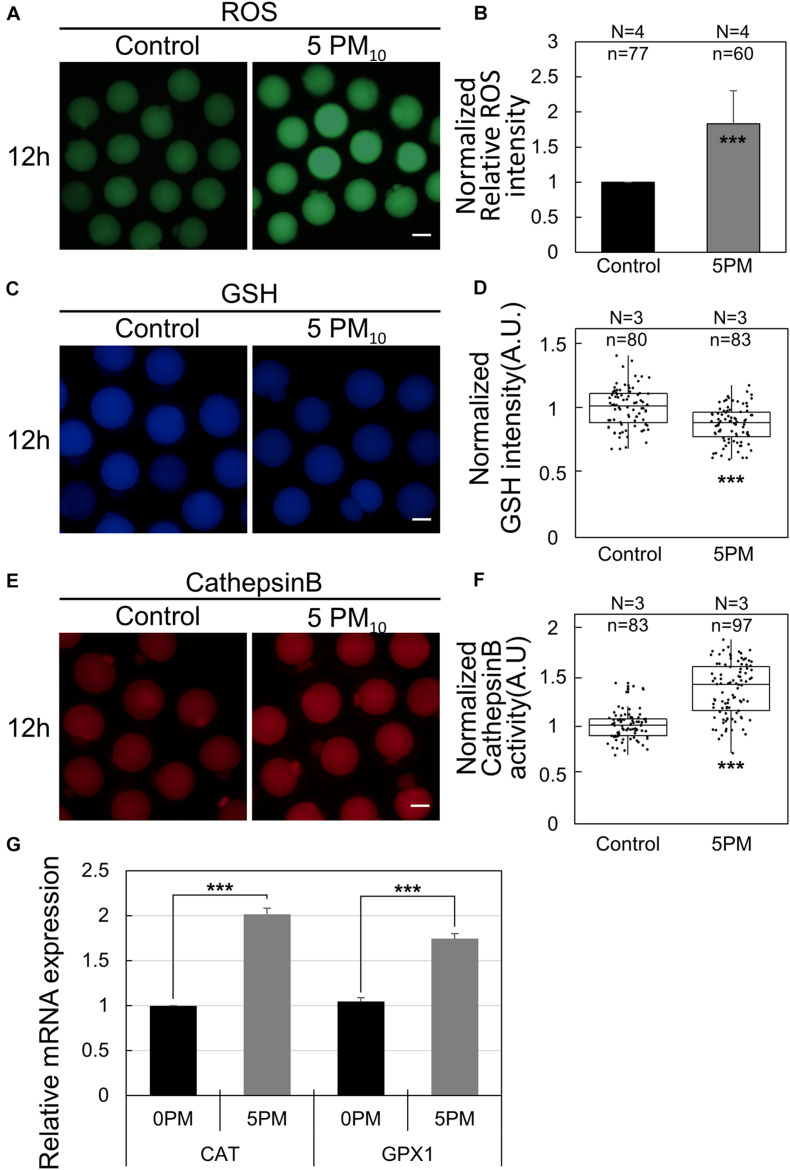
PM_10_ treatment effects on ROS level, GSH level, and cathepsin B activity during mouse oocyte maturation. **(A,C,E)** Effect of PM_10_ exposure on ROS generation **(A)**, GSH levels **(B)**, and cathepsin B activity **(E)**. Oocytes were examined 12 h after maturation. Scale bar = 50 μm. **(B,D,F)** Normalized relative intensity of ROS **(B)**, GSH **(D)**, and cathepsin B activity **(F)** Experimental replicates (N) and *n* values are indicated. **(G)** Relative mRNA expression of intracellular antioxidant enzyme-related genes at 12 h after meiotic resumption. Experiments were performed in triplicate and replicated three times with similar results; 20 oocytes were used for each repetition. ***P < 0.001.

To elucidate how PM induces defects in mouse oocyte maturation, we evaluated intracellular ROS generation, GSH levels, and cathepsin B activity in control and PM_10_-treated oocytes after 12 h of meiotic resumption (Figure 5A). ROS levels were determined by performing a DCFH fluorescence reaction. The relative fluorescence intensity of ROS was significantly higher in the PM_10_-treated oocytes than in control oocytes (Figures 5A,B). In contrast, normalized GSH fluorescence intensity was significantly lower in the PM_10_-treated group than in the control (Figures 5C,D). Finally, activated cathepsin B was analyzed, with the normalized results showing a significant increase in PM_10_-treated oocytes compared with control oocytes (Figures 5E,F). Real-time RT-PCR of oocytes subjected to 12 h of PM_10_ demonstrated that major intracellular antioxidant enzyme-related genes (*Cat* and *Gpx1*) were significantly increased in the PM_10_ treatment group compared with control (Figure 5G).

### PM_10_ Exposure Leads to Mitochondrial Dysfunction and Mitochondrial ROS Accumulation

**FIGURE 6:**
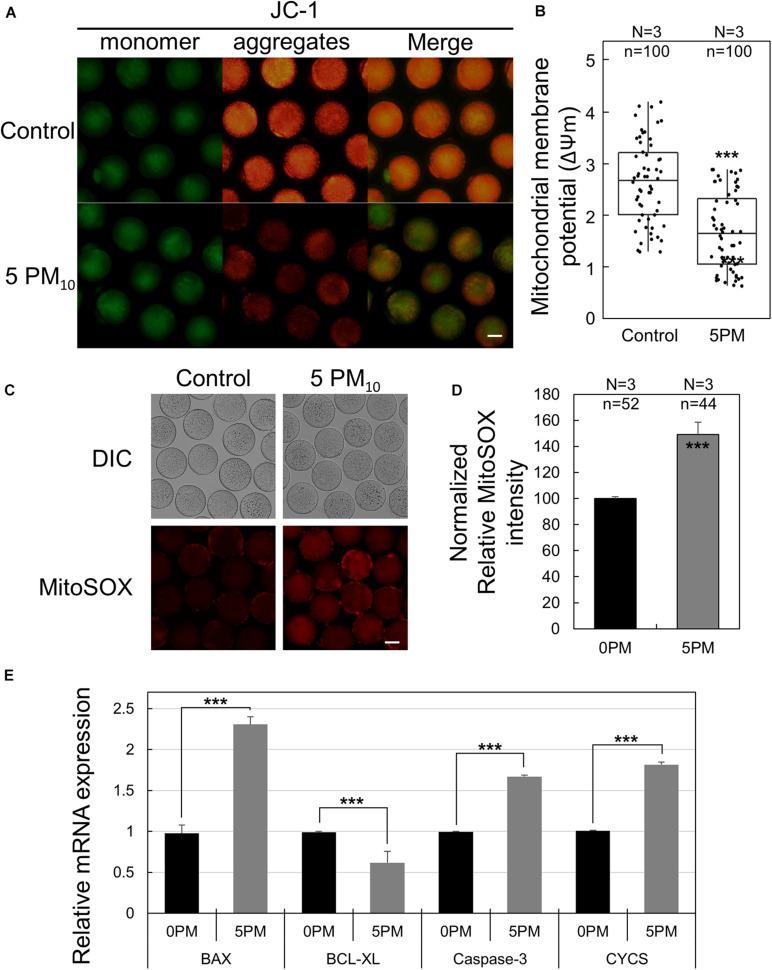
Effect of PM_10_ exposure on mitochondrial dysfunction and oxidative stress. **(A)** JC-1 staining to determine mitochondrial membrane potential. Oocytes were stained 12 h after maturation. Scale bar = 50 μm. **(B)** Mitochondrial membrane potential levels were determined by measuring ΔΨm (red/green). **(C)** Mitochondrion-specific superoxide was detected by MitoSOX red staining in the zona-free oocytes. Scale bar = 40 μm. **(D)** Relative MitoSOX fluorescent intensity. Experimental replicates (N) and *n* values are indicated in **(B)** and **(D)**. **(E)** Relative mRNA expression of the anti-apoptotic gene *BCL-XL*, the pro-apoptotic genes *BAX* and caspase3, and the mitochondrial apoptotic gene *CYCS* using a matured oocyte from the indicated groups. Experiments were performed in triplicate and replicated three times with similar results; 20 oocytes were used for each repeat. ***P < 0.001.

Because intracellular oxidative stress generally induces mitochondrial dysfunction, the mitochondria membrane potential—an indicator of oocyte cytoplasmic maturation—was evaluated by JC-1 staining. As shown in Figure 6A, the high membrane potential signal (red; J-aggregates) was markedly lower in the PM_10_ exposure group than in the controls; however, no difference was observed in terms of J-monomer signal (green). ΔΨm (ratio of J-aggregates/J-monomer) was significantly reduced in the PM_10_-treated oocytes compared with controls (Figure 6B). To measure mitochondrial ROS levels, we carried out MitoSOX red staining, which is an indicator of mitochondrion-specific superoxide expression generated by ATP production. The relative fluorescence intensity of MitoSOX red was significantly increased in the PM_10_-exposed oocytes than in the controls (Figures 6C,D). Taken together, these results show that the PM_10_ treatment induces mitochondrion-derived superoxide dysfunction as well as intracellular ROS generation during oocyte maturation. Real-time RT-PCR of 12-h PM_10_-exposed oocytes demonstrated that pro-apoptosis-associated genes (*Bax* and *Casp3*) and mitochondrial apoptosis-related gene (*Cycs*) were significantly upregulated, while the anti-apoptosis-associated gene *Bcl2l1* was significantly downregulated in the PM_10_ treatment group compared with control (Figure 6E).

## Discussion

Atmospheric PM (particulate matter; also termed fine dust) pollution is associated with adverse health effects in humans. Previous studies have shown that PM exposure can lead to infertility by disrupting normal reproductive processes such as spermatogenesis, fertilization, and trophoblast invasion, as well as angiogenesis during pregnancy (Perin et al., 2010; van den Hooven et al., 2012; Soto et al., 2017; Jurewicz et al., 2018; Familari et al., 2019), in addition to its adverse effects on fetal health (Veras et al., 2010). Female infertility rates have been increasing and atmospheric PM is one of the major contributing factors. Indeed, in human IVF cycle data, the association between increased ambient concentrations of PM and reduced pregnancy rates suggests that PM adversely affects the female reproductive system (Choe et al., 2018). PM exposure in female mice was also found to induce cellular damage in granulosa cells and oocytes following induction of abnormal ovarian ultrastructure.

Although it has been previously shown that PM pollution impacts the reproductive system, several factors remain unclear, including the effect of PM on female meiosis, occurring in the ovary *in vivo*, and the specific mechanism underlying the toxic phenotype. Therefore, the present study was conducted to investigate the toxic effect of PM_10_ on female reproductive cells during meiotic division (oocyte maturation). We found that cytokinesis decreased in PM_10_-exposed oocytes in a dose-dependent manner with increased abnormal PB formation (large-sized PBs such as those seen at the two-cell stage or a PB segregated into two). The ratio of normal MII oocytes also decreased even though cytokinesis occurred. Furthermore, PM_10_-treated oocytes failed to activate after SrCl_2_ treatment and second PB extrusion. Thus, PM_10_-treated oocytes fail to undergo fertilization and further embryonic development.

The activity of MPF, a complex of cyclin B1 and CDK1, is a key regulator of MI–MII transition during meiosis (Doree and Hunt, 2002; Adhikari et al., 2012). MPF levels increase after meiotic resumption and are maintained during metaphase. MI to ATI transition is permitted after MPF levels are reduced via cyclin B1 degradation (Josefsberg et al., 2001). Here, we found that PM exposure induces cell cycle arrest in maturing oocytes by blocking the degradation of cyclin B1. Similarly, evidence regarding alteration of cell cycle by PM exposure also supports the PM-induced arrest of G2/M phase and regulation of spindle organization (Longhin et al., 2013; Wu et al., 2017). Therefore, exposure to PM can arrest oocyte maturation at the metaphase stage and may regulate maturation such as spindle organization.

During meiosis, cell cycle arrest results from failure in formation of the barrel-shaped bipolar spindle and the spindle assembly checkpoint, which is involved in oocyte MI–ATI transition, DNA damage response, apoptosis, etc. (Wei et al., 2010; Palou et al., 2017; Ruan et al., 2018). During the early stage of meiotic spindle formation, the aMTOC gathers at the spindle pole and initiates microtubule assembly. Here, PM exposure resulted in the scattering of pericentrin in oocytes as they failed to accurately localize. Formation of the barrel-shaped spindle was thus disrupted and abnormal tubulin formation significantly increased. Abnormal spindle formation fails to capture the chromosomes and align at the equatorial plate of the oocyte cytoplasm, delaying cytokinesis (Lee et al., 2018). Subsequently, the spindle assembly checkpoint may activate or block meiosis at the pre-MI stage (Marangos et al., 2015). Concurrent with chromosome misalignment, DNA damage and early apoptosis rates increased in PM-exposed oocytes. Due to the toxic effects of PM, cell cycle arrest blocks subsequent pathways, eventually decreasing MPF levels via cyclin B1 degradation following inactivation of the anaphase-promoting complex/cyclosome.

In the present study, intracellular ROS levels increased during oocyte maturation in the presence of PM. Although ROS is reported to play an important role in mammalian oocyte maturation and embryo development (Kala et al., 2017), studies have shown that PM induces increased ROS generation, which leads to mitochondrial damage within various cells, thereby increasing the intracellular inflammatory response (Ovrevik et al., 2015; Liu et al., 2020). There is a delicate balance in the mitochondria between ROS generation and scavenging to maintain ROS homeostasis (Pan et al., 2009). A complex antioxidant defense system within cells relies on endogenous and non-enzymatic antioxidants to scavenge free radicals, reducing their damaging effects to important biomolecules; representative antioxidant enzymes include catalase, glutathione peroxidase 1 (GPx1), and superoxide dismutase (He et al., 2017). Catalase, a common antioxidant enzyme present in almost all living tissues that utilize oxygen, efficiently breaks down millions of H_2_O_2_ molecules per second. Catalase regulates ROS levels in oocytes through their transition to embryos via its antioxidant enzymatic activity (Cetica et al., 2001; Park et al., 2016). GPx, an important intracellular enzyme that breaks down H_2_O_2_ into water, is a protective enzyme that is central to GSH synthesis and was shown to be present in mature MII mouse oocytes as well as in failed-to-fertilize human MII oocytes (El Mouatassim et al., 1999). Here, *CAT* and *GPX1* mRNA levels were significantly elevated by PM. These results indicate that the PM-induced increase in ROS levels during oocyte maturation enhanced the expression of antioxidant enzymes. Moreover, PM-induced ROS generation in the mitochondria resulted in various types of damage during oocyte maturation; indeed, the expression of mitochondrial apoptosis-related genes, such as *Cycs, Bax, Bcl2l1*, and *Casp3*, was confirmed. Mitochondrial cytochrome *c* release occurs during DNA fragmentation, while caspase-3 activation is correlated with the release of cytochrome *c* from local mitochondria and can be prevented by BCL-2 overexpression (Garrido et al., 2006). Therefore, excessive exposure to PM inhibits oocyte maturation; moreover, the results of the present study indicate that various types of PM generated by environmental pollutants, such as vehicle exhaust emissions, directly affect mammalian germ cells.

PM is a mixture of diverse components found in the air that can be categorized according to its source: coal burned for electricity, industrial fuel, manufacturing processes, oil refining, and motor vehicles. The fine dust employed in the present study was collected from tunnel walls; therefore, the effect of PM from vehicles and urban dust on female meiosis was studied. As the present study is the first to investigate PM exposure during mammalian oocyte maturation, various doses of PM were evaluated, in particular, the 5 mg/mL concentration. However, the material employed and the method of PM induction differ, including where/how to capture the PM, sample dilution, treatment according to cell type, etc. Therefore, future experiments should be conducted with caution.

## Conclusion

In conclusion, we demonstrated the toxic effects and mechanism of PM_10_ exposure in maturing mouse oocytes. PM_10_ exposure induces cell cycle arrest during MI-ATI transition by blocking MPF degradation. Defects in spindle formation and chromosome alignment occurred after the PM_10_-induced DNA damage and apoptosis. This may potentially result in genetic disorders as a consequence of asymmetric chromosome division. ROS accumulation and mitochondrial dysfunction were also seen following PM_10_ exposure. Thus, exposure to PM during mammalian oocyte maturation disrupts oocyte quality and can lead to female infertility by inhibiting developmental potential. Further experiments are needed to investigate the toxic effect of PM on IVF, early embryogenesis, and implantation.

## Data Availability Statement

The original contributions presented in the study are included in the article/Supplementary Material, further inquiries can be directed to the corresponding author.

## Ethics Statement

The animal study was reviewed and approved by Animal Research Committee of Korea Research Institute of Bioscience and Biotechnology (KRIBB-AEC-19126).

## Author Contributions

J-SK conceived the project. Y-JJ and S-BY designed the study and conducted experiments. J-SK, Y-JJ, and S-BY wrote and revised the manuscript. All authors contributed to analyze the data and reviewed the manuscript.

## Conflict of Interest

The authors declare that the research was conducted in the absence of any commercial or financial relationships that could be construed as a potential conflict of interest.
