# Supplementary material for: Particulate Matter Exposure During Oocyte Maturation: Cell Cycle Arrest, ROS Generation, and Early Apoptosis in Mice
Source: Front Cell Dev Biol. 2020 Nov 26;8:602097. doi: 10.3389/fcell.2020.602097 (PMC7726243; doi:10.3389/fcell.2020.602097)
Supplement: Supplementary file 1 [file Data_Sheet_1.PDF]

Supplementary Material

Supplementary Table 1. Primer sequences used for qPCR.

Gene	Primer sequences	GenBank accession no.	Product size (bp)
CAT	F: 5’- CCTCGTTCAGGATGTGGTTT -3’<br>R: 5’- TCTGGTGATATCGTGGGTGA-3’	NM_009804.2	130
GPx1	F: 5’- CAAGTACGTCCGACCTGGTG -3’<br>R: 5’- GTCGGTCATGAGCGCAGT -3’	NM_001329527.1	151
Bax	F: 5’- AGGCCTCCTCTCCTACTTCG -3’<br>R: 5’- CTCAGCCCATCTTCTTCCAG -3’	NM_007527.3	104
Bcl-XL	F: 5’- CCTTCAGGCCTCTCTCTCCT-3’<br>R: 5’- CCAGCAGCTCCTCACACATA-3’	NM_001289716.1	179
Caspase-3	F: 5’- GGGCGTGTTTCTGTTTTGTT -3’<br>R: 5’- TTGAGGTAGCTGCACTGTGG -3’	NM_001284409.1	138
Cycs	F: 5’- CACCGACACCGGTACATAGG -3’<br>R: 5’- TAATTCGTTCCGGGCTGGTC -3’	NM_007808.5	108
GAPDH	F: 5’-ACCATCTTCCAGGAGCGAGA-3’<br>R: 5’-GGGCCATCCACAGTCTTCTG-3’	NM_001289726.1	350

Supplementary Movie 2. Time-lapse movie of an oocyte injected with Cyclin B1-GFP and H2B-mCherry of control oocyte (Left: GFP+mCherry; right: GFP+mCherry+DIC merged). Images were taken 0-720 min after meiotic resumption. The frame interval is 5 min and the total length of the movie is 12 h (720 min). Cyclin B1-GFP intensity was decreased in control oocyte at the timing of the MI-ATI transition. Gray; DIC, green; tubulin, red; chromatin. Scale bar: 20μm.

Supplementary Movie 2. Time-lapse movie of an oocyte injected with Cyclin B1-GFP and H2B-mCherry of control oocyte (Left: GFP+mCherry; right: GFP+mCherry+DIC merged). Images were taken 0-720 min after meiotic resumption. The frame interval is 5 min and the total length of the movie is 12 h (720 min). Cyclin B1-GFP intensity was decreased in control oocyte at the timing of the MI-ATI transition. However, intensity of Cyclin B1-GFP was stable in PM exposure oocyte in maturing oocyte. Gray; DIC, green; cyclin B1, red; chromatin. Scale bar: 20μm.

Supplementary Movie 3. Time-lapse movie of an oocyte injected with tubulin-GFP and H2B-mCherry of control oocyte (Left: GFP+mCherry; right: GFP+mCherry+DIC merged). Images were taken 0-390 min after meiotic resumption. The frame interval is 5min and the duration of the movie is 6.5h (390min). Gray; DIC, green; tubulin, red; chromatin. Scale bar: 20μm.

Supplementary Movie 4. Time-lapse movie of an oocyte injected with tubulin-GFP and H2B-mCherry of control oocyte 5 PM_{10} treated oocyte (Left: GFP+mCherry; right: GFP+mCherry+DIC merged). Images were taken 0-390 min after meiotic resumption. The frame interval is 5min and the duration of the movie is 6.5h (390min). Gray; DIC, green; tubulin, red; chromatin. Scale bar: 20μm.
